# Notes and News

**Published:** 1894-05-05

**Authors:** 


### Notes and News.

Collected and Communicated.

MEETINGS OF THE WEEK.

Children's Hospital, Great Ormond Street, Festival Dinner, Hotel Metropole, Saturday, May 5th, 7.30 p.m.
East London Hospital for Children, Festival Dinner, Hotel Metropole, Monday, May 7th, 7.30 p.m.
City of London Truss Society, Festival Dinner, Albion, Aldersgate Street, Tuesday, May 8th, 6.30 p.m.

It is reputed that cholera of a most malignant type has broken out in Constantinople, and is spreading with alarming rapidity. The disease had been prevalent in the poorer and most crowded quarters, but it has now invaded the districts in which the richer classes reside.

The centenary of the Sunderland Infirmary, which was founded in May, 1794, will be reached this month. This particular epoch in the history of the institution will be marked by the completion of the convalescent home at Heatherdene. The latter establishment, so far, has only provided accommodation for women and children patients. The proposed extension deals with providing a wing for male inmates. For this excellent object a demonstration will take place on June 16th in favour of the necessary funds, whence it is hoped some—if not all—of the £3,500 required for the purpose will be forthcoming.

The annual report of the Metropolitan Asylums Board shows that the number of patients in the various fever hospitals has considerably increased during the year. In 1891 they amounted to 7,873, in 1893 to 21,129. Whilst the law demands that fever patients should be removed to proper places of treatment, the Board is unable to meet the demand for accommodation. This arises, it is stated, principally from the difficulty experienced in finding sites for new hospitals. The opposition encountered on the choice of any neighbourhood and the locality in which to erect such an institution is most formidable and difficult to overcome, and causes much regrettable de. lay, where patients in hundreds are being treated in their own homes, and are the means of a great spread of disease.

University College, Liverpool, has become the recipient of munificent gifts from Mr. Henry Tate and Lord Derby. The former has presented books to the value of £5,000, and Lord Derby has given £10,000 to endow a chair of Anatomy.

A most interesting exhibition is now open at the Imperial Institute, closing, we believe, on Saturday. This is the Amateur Exhibition of Art, opened by the Duchess of Albany on Wednesday. Many of the Royal family were amongst the exhibitors. The Queen's collection of portraits by John Russell, R.A., is very interesting, and it has been arranged and catalogued by Dr. Williamson. Mr. Probert has lent a collection of fine miniatures, and there are, of course, many interesting exhibits by living artists. Great pains has been taken to render the exhibition a success, and we hope that financially it will add largely to the funds of the three charities it was inaugurated to benefit, namely, the East London Nursing Society, the East-end Mothers' Home, and the Parochial Mission Women's Fund.

Curious notions on the subject of quackery would appear to prevail amongst some members of the New York Medico-Legal Society. One of these gentlemen has recently been advocating the adoption of certain modifications in the Medical Practice Act for the benefit of "Christian scientists," clairvoyants, and others, giving as his opinion that the legislature should no more have the power to dictate the methods of a man's cure than a railway company should compel people to use its trains. He adduces by way of argument that one of his daughters had been cured by a clairvoyant, while a qualified physician had done no good, whilst he laid the death of a second at the door of another incompetent doctor. Another Bill has been introduced into the Assembly providing, says a New York contemporary, "That any persons may freely contract for the services of whomsoever he considers competent to alleviate suffering or prevent or heal disease, and that it shall be lawful for persons so employed to attend, treat, nurse, and endeavour to heal the employing patient, and receive compensation therefor, subject to the laws of the State relative to malpractice."

The editor of *The Young Man* and *The Young Woman* is arranging another great holiday tour for young men and women. It will be held at Grindelwald in August and September, and the opening address is to be delivered by Sir B. W. Richardson on "How to Make the Most of Life." Sir Robert Ball will deliver two lectures, and Mr. Edmund Gosse will give "A Talk about Books." Twice a week there will be concerts and social receptions. Miss Mary Davies hopes to be able to sing at four concerts. Parties will leave London every Tuesday and Friday from June 29th to September 14th, and a twelve days' Swiss holiday is offered for ten guineas, including second-class return ticket from London (first-class on boat), and full hotel accommodation. All tickets will be available to return any time within forty-five days. Further particulars can be obtained by sending a stamped addressed envelope to Mr. F. A. Atkins, 2, Amen Corner, London, E.C. Under proper management such an opportunity to travel economically and comfortably will prove of the utmost benefit. Provided the mental part of the programme is to be carried out in the evening, it will doubtless be much appreciated also.

